# Optimization of *Clostridium beijerinckii* semi-solid fermentation of rape straw to produce butyric acid by genome analysis

**DOI:** 10.1186/s40643-024-00742-y

**Published:** 2024-02-14

**Authors:** Hui Kou, Jia Zheng, Guangbin Ye, Zongwei Qiao, Kaizheng Zhang, Huibo Luo, Wei Zou

**Affiliations:** 1 College of Bioengineering, Sichuan University of Science & Engineering, No.1 Baita Road, Sangjiang District, Yibin, 644005 Sichuan China; 2 School of Laboratory Medicine, North Sichuan Medical College, Nanchong, 637007 Sichuan China; 3 Wuliangye Yibin Co., Ltd., Yibin, 644000 Sichuan China; 4 Liquor Brewing Biotechnology and Application Key Laboratory of Sichuan Province, Sichuan University of Science & Engineering, Yibin, 644005 Sichuan China

**Keywords:** Butyric acid, *Clostridium beijerinckii*, Semi-solid non-sterile fermentation, Rape straw, Genome annotation

## Abstract

Butyric acid is a volatile saturated monocarboxylic acid, which is widely used in the chemical, food, pharmaceutical, energy, and animal feed industries. This study focuses on producing butyric acid from pre-treated rape straw using simultaneous enzymatic hydrolysis semi-solid fermentation (SEHSF). *Clostridium beijerinckii* BRM001 screened from pit mud of Chinese nongxiangxing baijiu was used. The genome of *C. beijerinckii* BRM001 was sequenced and annotated. Using rape straw as the sole carbon source, fermentation optimization was carried out based on the genomic analysis of BRM001. The optimized butyric acid yield was as high as 13.86 ± 0.77 g/L, which was 2.1 times higher than that of the initial screening. Furthermore, under optimal conditions, non-sterile SEHSF was carried out, and the yield of butyric acid was 13.42 ± 0.83 g/L in a 2.5-L fermentor. This study provides a new approach for butyric acid production which eliminates the need for detoxification of straw hydrolysate and makes full use of the value of fermentation waste residue without secondary pollution, making the whole process greener and more economical, which has a certain industrial potential.

**Graphical Abstract:**

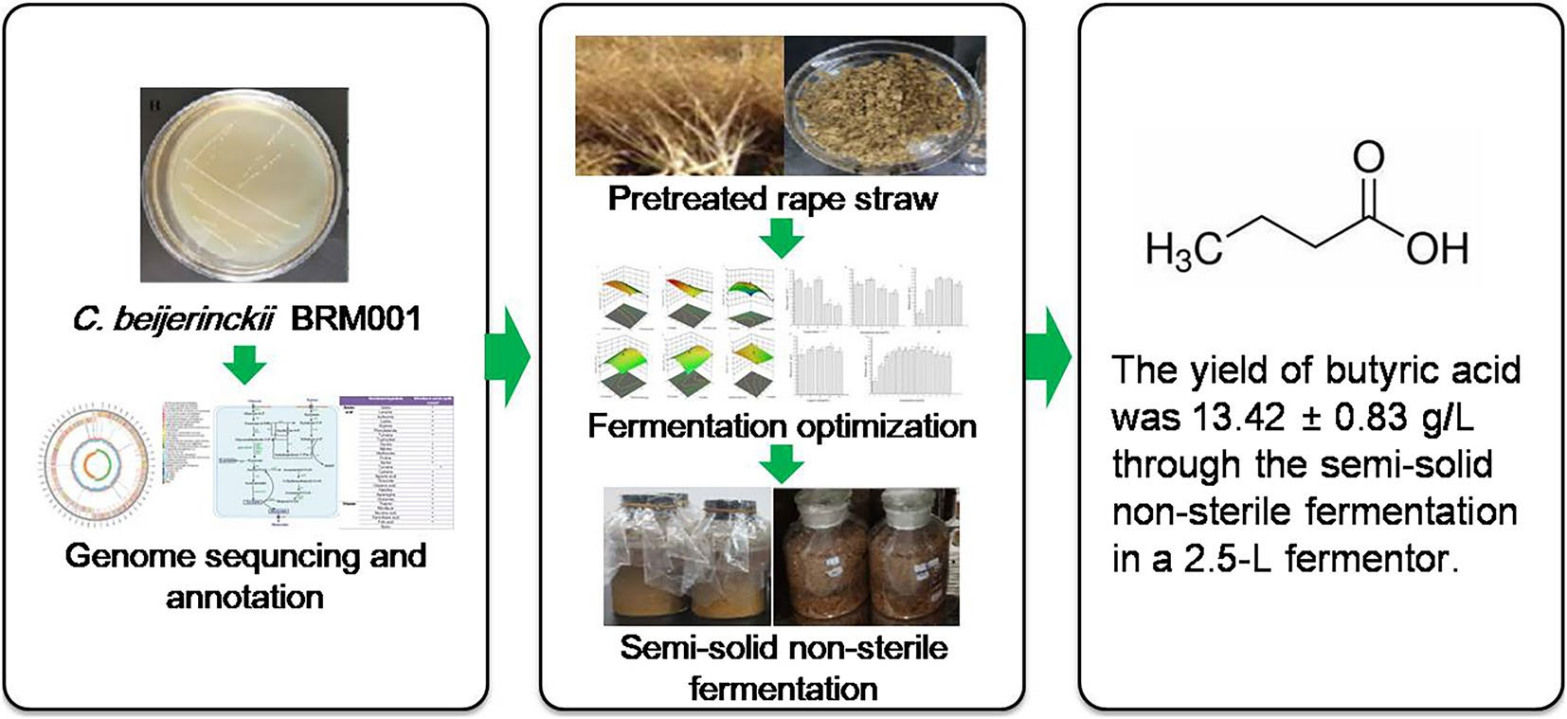

**Supplementary Information:**

The online version contains supplementary material available at 10.1186/s40643-024-00742-y.

## Introduction

Butyric acid (CH_3_CH_2_CH_2_COOH) is a short-chain volatile fatty acid with important applications in the chemical, food, pharmaceutical, energy, and animal feed industries (Luo et al. 2018; Dwidar et al. 2013). At present, the industrial production of butyric acid is mainly performed by petroleum-based chemical synthesis. However, microbial fermentation has attracted more attention due to its advantages in environmental protection, resource reuse, and sustainable development (Heng et al. 2021).

So far, due to the high substrate cost and low butyric acid concentration, the cost of butyric acid production by biological fermentation is still much higher than that by chemical synthesis (Luo et al. 2018). It is necessary to explore cost-effective substrates to reduce the production cost of bio-butyric acid. Many groups have begun to produce butyric acid from lignocelluloses (Ai et al. 2016; Chi et al. 2018; Fonseca et al. 2021; Liu et al. 2013). Rape is one of the main oil crops in China, and its planting area ranks first in the world (Meng et al. 2021). Rape straw contains a large amount of lignocellulose, which could be degraded into fermentable sugars for microorganisms to produce butyric acid. However, the production of butyric acid from rape straw still faces many challenges, such as low yield, and the toxic inhibitors released during the pretreatment process lead to poor cell growth, call for detoxification, or require specific equipment, and the treatment of fermentation waste is not considered, hindering industrial application (Fonseca et al. 2021; Huang et al. 2016).

*Clostridium beijerinckii* is a facultative anaerobe that can use a wide range of carbon sources. It is easy to cultivate and it can use glucose and xylose as representative lignocellulosic sugars (Birgen et al. 2018). *C. beijerinckii* has been used to produce butyric acid in a few studies (Fonseca et al. 2021; Tian et al. 2019), and the use of *C. beijerinckii* to produce butyric acid has great development prospects.

Liquid fermentation of rape straw to produce butyric acid is a step-by-step hydrolysis fermentation process. The hydrolysis and saccharification of straw and the fermentation of straw hydrolysate are carried out in two reactors in turn (Thirmal and Dahman, 2012). The disadvantages of liquid fermentation include high equipment cost, low production efficiency, and high production energy consumption (Li et al. 2016). Only the straw liquid is used as the carbon source to configure the medium for fermentation to produce butyric acid, and the residue of the hydrolysate will cause secondary pollution to the environment. Thus, simultaneous saccharification and fermentation (SSF), which solves the problem of feedback inhibition of cellulose hydrolysates and the filter of residue, can be used to produce butyric acid from rape straw (Chi et al. 2018).

In this study, *C. beijerinckii* BRM001 isolated from the pit mud of Chinese nongxiangxing baijiu was used to produce butyric acid from rape straw. The whole genome of *C. beijerinckii* BRM001 was sequenced and annotated. Through genome analysis of BRM001, optimization of the fermentation medium and the process was carried out. Then, non-sterile SSF was performed and the scale was increased to a 2.5-L fermentor. The aim of this study was to develop a high-yield, green, and economical process for the production of butyric acid by lignocellulose fermentation.

## Materials and methods

### Preparation of rape straw hydrolysate

Rape straw was collected from a farmland in Cuiping District, Yibin City, Sichuan Province, China in May 2021. The collected rape straw, including the main stalks and branches, were crushed together, dried, and then filtered with a 20 mesh sieve. Pretreatment and enzymatic hydrolysis of straw were carried out using Bai’s method (Bai et al. 2019), and the concentration of treatment solution, pretreatment temperature, enzymatic hydrolysis time, enzymatic hydrolysis temperature, citric acid buffer concentration, and other factors were improved as follows: rape straw was mixed with 1.1% NaOH (w/v) at a solid-to-liquid ratio of 1:10 and allowed to stand at room temperature (about 25 °C) for 48 h, rinsing with tap water to neutral drying, addition of Novozymes cellulase Cellic CTec3 HS 0.1% (enzyme activity 90 FPU/mL), a citric acid concentration of 0.01 M, and standing for enzymatic hydrolysis at room temperature (about 25 °C) for 48 h, followed by filtration; the clear supernatant was taken as rape straw hydrolysate.

### Microorganism and culture media

*C. beijerinckii* BRM001 was isolated from pit mud in a Nongxiangxing Baijiu factory by the Reinforced Clostridial Medium (RCM) in the early stage. The Nongxiangxing Baijiu factory is located in Yibin City, Sichuan Province, China. BRM001 has been preserved in the Guangdong Provincial Microbial Culture Collection Center under preservation number GDMCC No: 62710.

The percent purity and company of the reagents/chemicals used in this study are shown in Additional file 1: Tables S1 and S2.

Activation medium (RCM) contained 5 g/L glucose, 5 g/L NaCl, 5 g/L yeast extract, 10 g/L peptone, 3 g/L sodium acetate, 10 g/L beef extract, 1 g/L soluble starch, and 0.5 g/L L-cysteine hydrochloride, pH 6.8, and was sterilized at 121 °C for 20 min (Heng et al. 2021).

Synthetic medium (SM) was prepared by classical methods and contained glucose, NH4^+^, Na^+^, K^+^, Cl^−^, SO_4_^2−^,and PO_4_^3−^ (Zhang and Greasham, 1999). Referring to the synthetic medium of Popoff (Popoff 1984), the SM used in the present study contained (final concentrations) 15 g/L glucose,0.9 g/L NaCl, 0.7 g/L K_2_HPO_4_,0.7 g/L KH_2_PO_4_, 3 g/L Na_2_SO_4_, 10 g/L ammonium acetate, and 0.5 g/LL-cysteine salt, pH 7.0 ± 0.5, and was sterilized at 120 °C for 20 min. After filtration and sterilization, 5% key nutrient factor solution and 0.5% metal ion solution were added. The metal ion solution was prepared based on the formula of Zhang et al (2011). The nutrient solution included 0.05 g/L biotin and 5 g/L tryptophan.

The principle of fermentation medium preparation is to replace the glucose in the synthetic medium with straw saccharides and add trace yeast powder to replace some key nutritional factors, as follows:

Basic fermentation medium 1 was prepared as follows. Straw saccharification liquid (hydrolysate obtained after 10 g/L straw pretreatment) was obtained by filtration of the solution prepared as described in “Preparation of rape straw hydrolysate” section, containing (final concentrations) 0.9 g/L NaCl, 1 g/L yeast powder, 0.7 g/L H_2_KPO_4_, 0.7 g/L HK_2_PO_4_, 10 g/L CH_3_COONH_4_, and 0.5 g/L L-cysteine hydrochloride, pH 6.8, and was sterilized at 121 °C for 20 min, followed by the addition of 5% nutrient solution and 0.5% metal ion solution.

Basic fermentation medium 2 contained (final concentrations) 10 g/L straw, 0.01 M citric acid (stock solution prepared using tap water), 0.9 g/L NaCl, 1 g/L yeast powder, 0.7 g/L H_2_KPO_4_, 0.7 g/L HK_2_PO_4_, 10 g/L CH_3_COONH_4_, and 0.5 g/L L-cysteine hydrochloride, pH 6.8, and was sterilized at 121 °C for 20 min, followed by the addition of 5% nutrient solution and 0.5% metal ion solution.

### Genome sequencing, assembly, and annotation

De novo sequencing of the *C. beijerinckii* BRM001 genome was performed using the Illumina HiSeq combined with PacBio sequencing method by Shanghai Meiji Biomedical Technology Co., Ltd. After quality inspection, high-quality data were used to construct the database.

SOAPdenovo2 was used to assemble the contigs and scaffolds (Luo et al. 2012). Third-generation sequence assembly was performed using unicycler v0.4.8 (Wick et al. 2017), and sequence correction was performed using pilon v1.22. Glimmer (Delcher et al. 2007), GeneMarkS, and Prodigal were used to predict the coding sequences in the genome. The presence of tRNA in the genome was predicted using tRNAscan-SE v2.0 (Chan and Lowe, 2019). Barrnap (https://github.com/tseemann/barrnap) was used to predict the presence of rRNA in the genome. Annotation was performed using the National Center for Biotechnology Information (NCBI) Prokaryotic Genome Annotation Pipeline (Tatusova et al. 2016). The metabolic pathways were annotated by KAAS combined with the Kyoto Encyclopedia of Genes and Genomes (KEGG) database (Kanehisa et al. 2016). Using Circos software to draw a genome circle diagram of a single sample (Krzywinski et al. 2009), new information circles, such as ncRNA, GI, and Prophage, can be customized and added to the same circle or to different circles.

### Butyric acid fermentation

Liquid-state fermentation (also known as stepwise saccharification and fermentation): *C. beijerinckii* BRM001 seed liquid (5 mL) was inoculated into 100 mL basic fermentation medium 1, followed by anaerobic fermentation at 35 °C for 7 days.

Simultaneous enzymatic hydrolysis semi-solid fermentation (SEHSF): the enzymatic saccharification of straw and the fermentation of straw hydrolysates both occurred simultaneously in the same container, the process is simplified compared to saccharification in a saccharification vessel and then transfer to a fermentation reactor for fermentation*. C. beijerinckii* BRM001 seed liquid (5 mL) was inoculated into 100 mL basic fermentation medium 2, followed by anaerobic fermentation at 35 °C for 9 days (2 days of enzymatic saccharification and 7 days of fermentation). The content of butyric acid in fermentation broth was used to determine the best fermentation method.

Non-sterile SEHSF: *C. beijerinckii* BRM001 seed liquid was inoculated in non-sterile optimized fermentation medium, followed by fermentation under the optimized fermentation conditions.

### Optimization of butyric acid production of BRM001 based on genome analysis

KAAS annotation predicted that *C. beijerinckii* BRM001 had defects in the synthesis of some biomass components. The single factor experiment of SM was used to verify which biomass components lack will inhibit the growth of *C. beijerinckii* BRM001, and the effects of adding these biomass components to the fermentation medium on butyric acid production were explored. Then, the fermentation medium was optimized by response surface methodology (RSM) to improve butyric acid production.

The fermentation conditions of butyric acid production by *C.beijerinckii* BRM001 were optimized by a single factor experiment, and the value points of each factor were taken when the maximum value was reached. The single factors were temperature, inoculation amount, initial pH, liquid volume, and fermentation time. According to the results of our single factor experiment, the factors that had the greatest influence on the fermentation of *C.beijerinckii* BRM001 were selected to design an orthogonal experiment, and the optimal fermentation conditions were obtained.

To develop a low-cost, large-scale, applicable lignocellulose fermentation process for butyric acid production, considering the prospects of industrial application, we increased the volume of fermentation medium to 500 mL, 1000 mL, and 2500 mL without sterilization under the optimal fermentation conditions to produce butyric acid.

### Analytical determination and data processing

The content of butyric acid in fermentation broth was determined by GC with an external standard. GC conditions were as follows: an Agilent DB-WAX column (0.18 mm × 0.18 mm × 20 m); inlet temperature, 250 °C; FID detector temperature, 250 °C; carrier gas, hydrogen (99.999%); flow rate, 1 mL/min; split injection method; split ratio, 10:1; ramp-up procedure, initial temperature 80 °C, hold for 1 min, ramp up to 200 °C at 20 °C/min, then to 250 °C at 50 °C/min, hold for 5 min. This program could improve the separation effect and shorten the separation time (Blumberg and Klee 2001).

The straw saccharification liquid prepared under the optimal pretreatment conditions and enzymatic hydrolysis conditions was sent to the analysis and detection center of Sichuan University of Science & Engineering for detection. The carbohydrates in the saccharification liquid were detected by HPLC–MS (Xu et al. 2011), and the inhibitors in the saccharification liquid were determined by GC–MS and HPLC–MS.

The crude protein content was determined by the Kjeldahl nitrogen method with reference to GB/T 6432-2018. The determination of true protein was performed by T/NAIA 060-2021. The determination of acid-soluble protein was based on the standard NY/T 3801-2020.

RSM was performed using Design-Expert 8.0.6 (https://www.statease.com/software). The orthogonal experiment was performed using IBM SPSS Statistics 24 (https://www.ibm.com/support/pages/downloading-ibm-spss-statistics-28010). Origin (https://www.originlab.com/) was used to draw graphs and histograms, and Adobe Illustrator 2020 (https://www.adobe.com/cn/products/illustrator) was used to draw related graphs.

## Results and discussion

### Features of the hydrolysate of rape straw

Many kinds of carbon sources were detected in the hydrolysate of rape straw by HPLC–MS (Table 1), including glucose, xylose, fructose, mannose and other carbon sources. Therefore, the use of rape straw saccharification liquid as a carbon source for *C. beijerinckii* BRM001 fermentation is equivalent to co-fermentation of mixed sugars mainly composed of glucose and xylose. *C. beijerinckii* NCIMB 8052 has been reported to utilize glucose and xylose as representative lignocellulosic sugars (Chen and Blaschek 1999).

**Table 1:** Sugar components in rape straw hydrolysate

Species	Content (%)
Glucose	48.95
Xylose	22.61
Rhamnose	9.76
Arabinose	5.23
Fucose	3.66
Galactose	2.44
Mannose	1.83
Maltose	1.06
Sugar	0.32
Fructose	0.25
Cellobiose	0.16

Many substances that inhibit the growth of strains are produced during the preparation of straw hydrolysate (Cho et al. 2009). For example, when the toxicity limit is 1.8 m M, ferulic acid can destroy the hydrophobic point of *Clostridium* cells and increase membrane permeability, resulting in leakage of cell contents (Sánchez-Maldonado et al. 2011; Adeboye et al. 2014). When its concentration reaches a certain level, it will completely inhibit the growth of *C. beijerinckii* (> 0.9 g/L) (Liu et al. 2016). Quantitative detection of inhibitor concentrations in hydrolysate could determine whether it is necessary to perform detoxification, which is of great significance to improve fermentation efficiency. Through GC–MS and HPLC–MS detection, it was found that the hydrolysate of rape straw contained a variety of inhibitors, mainly including glacial acetic acid, ferulic acid, p-hydroxybenzaldehyde, syringaldehyde, 5-hydroxymethylfurfural, phenol, furfural, and acetyl syringone (Additional file 1: Table S3). Among them, the content of acetic acid was the highest, accounting for 0.992% of the saccharification liquid, followed by phenol, accounting for 0.079%, and the relative content of other inhibitors was low, accounting for ≤ 0.01%. Acetic acid is a precursor for the synthesis of butyric acid. The concentration of phenolic compounds generally reaches 1%, which has a significant inhibitory effect (Zhang et al. 2019). Therefore, detoxification was not required in this study.

### Genome sequencing and functional annotation of *C. beijerinckii* BRM001

#### Genome assembly and annotation

**Fig. 1:**
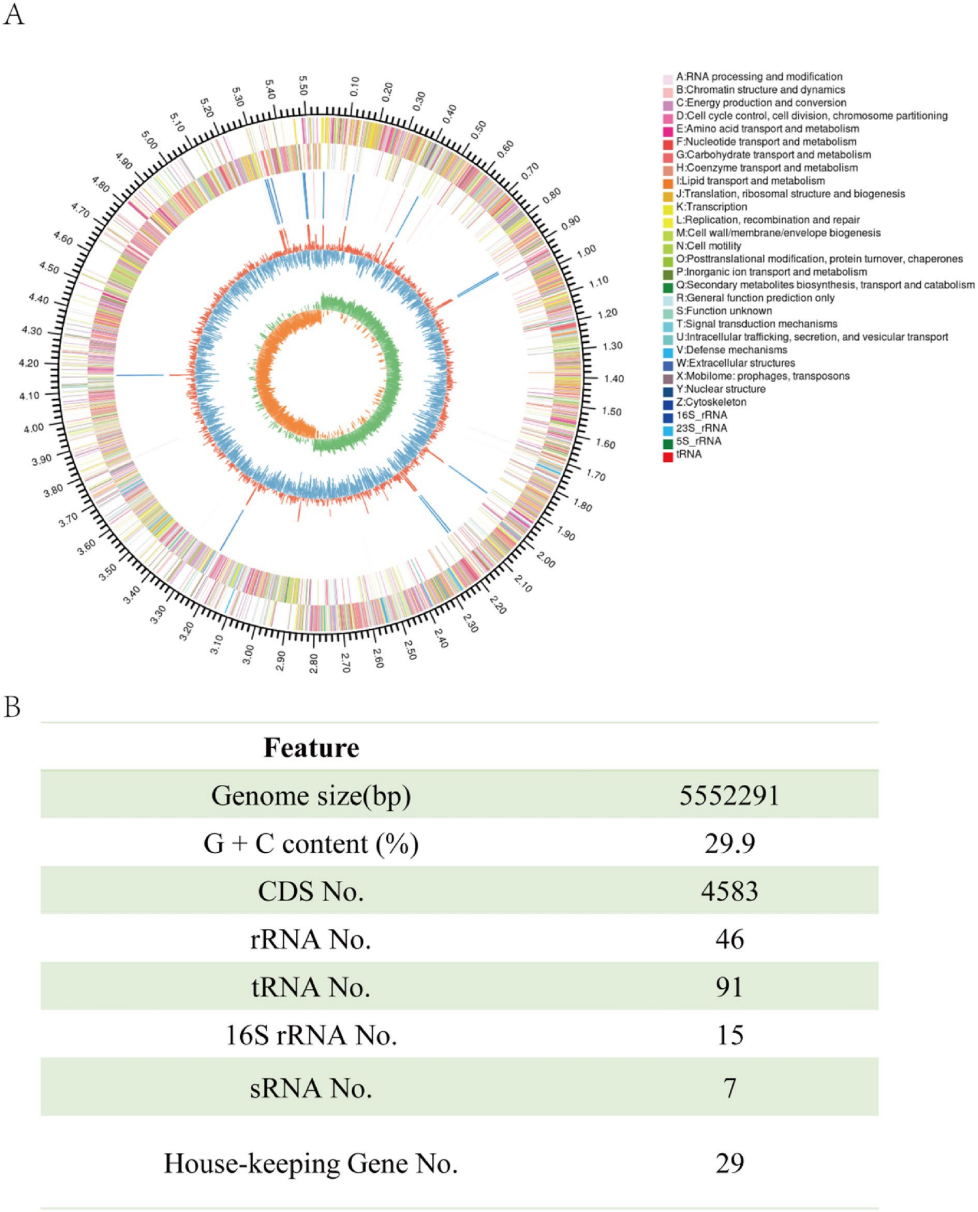
Basic characteristics of *C. beijerinckii* BRM001 genome (**A** Genome circle of *C. beijerinckii* BRM001, **B** Basic characteristics of the genome)

The whole genome sequence of *C. beijerinckii* BRM001 was determined by Illumina HiSeq combined with PacBio sequencing technology. *C. beijerinckii* BRM001 has only one chromosome and contains no plasmids. The genome length is 5,552,291 bp, the GC content is 29.9%, and the genome contains 4853 coding sequences. The genome contains 46 sets of 16S-23S-5S ribosomal RNA operons, 91 tRNA genes, and 20 tRNA gene types. The genome circle and basic characteristics of the genome are shown in Fig. 1. The assembled genome sequence was uploaded to the NCBI database, and the GenBank accession number is PRJNA940296.

According to the KAAS annotation of *C. beijerinckii* BRM001, a total of 2490 genes were assigned with KO numbers. The metabolic sub-systems with the most genes were carbohydrate metabolism (341) and membrane transport (271). In addition, amino acid metabolism (180) and vitamin and cofactor metabolism (154) also accounted for a large proportion.

#### Butyric acid biosynthesis pathway

To construct the butyric acid biosynthesis pathway, first, we chose the glucose and xylose, which were the two main components of rapeseed straw hydrolysate, as the carbon substrates. Second, the metabolic pathway maps were created through KAAS pipeline. The biosynthesis pathway of butyric acid from glucose and xylose in C. *beijerinckii* BRM001 were constructed by combined the related metabolic reactions together. These metabolic reactions were found in those KEGG pathway maps including ABC transporters (kegg map02010), Phosphotransferase system (PTS) (kegg map02060), Glycolysis/Gluconeogenesis (kegg map00010), Pentose and glucuronate interconversions (kegg map00040), Pyruvate metabolism (kegg map00620), and Butanoate metabolism (kegg map00650). Third, the butyrate transporter was annotated by Transporter Classification Database (TCDB).

**Fig. 2:**
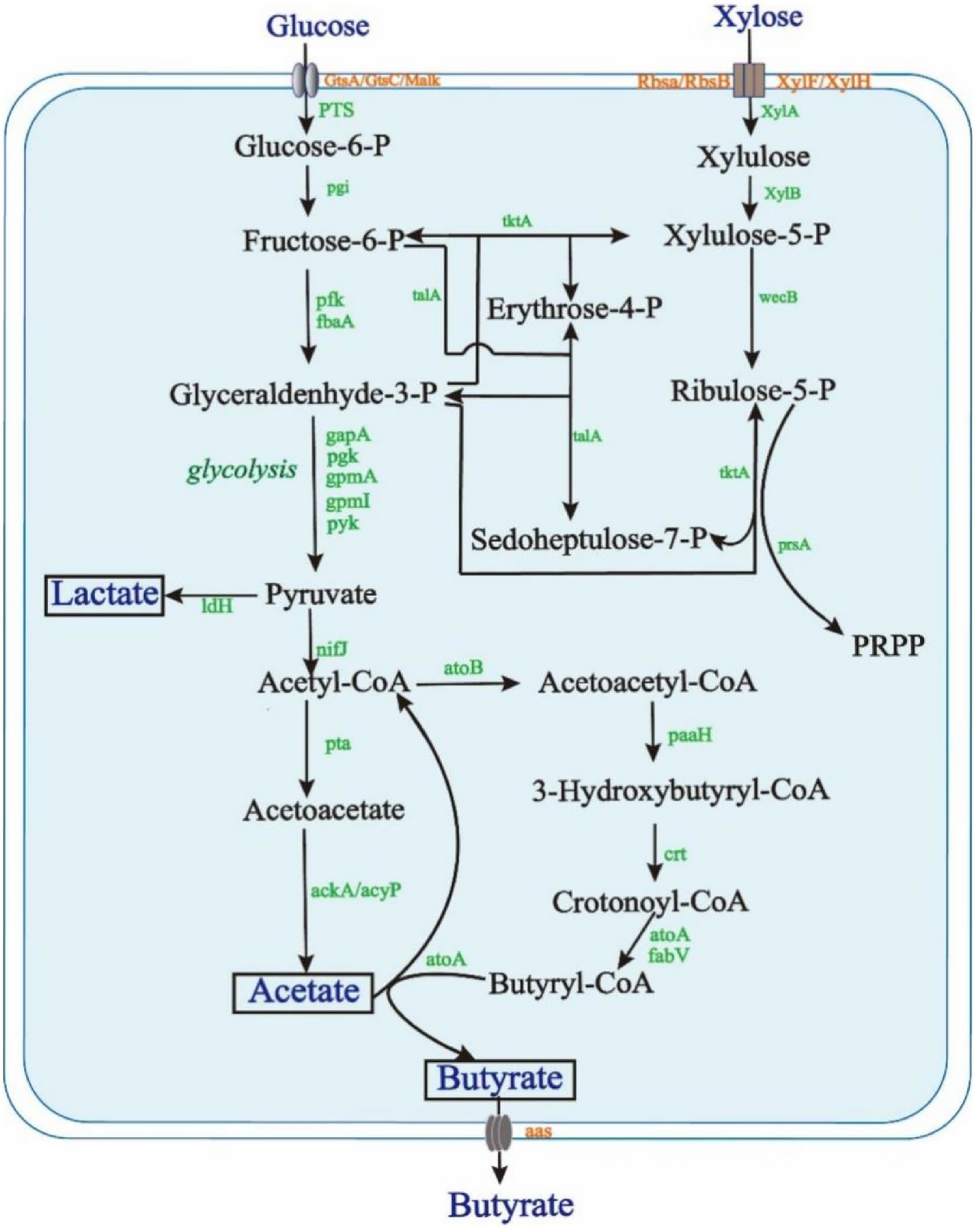
Metabolic pathways of butyric acid in BRM001. The local gene name and enzyme number corresponding to the gene name of each reaction step are shown in Additional file 1: Table S6

Therefore, the biosynthesis pathway of butyric acid from glucose and xylose in *C. beijerinckii* BRM001 was obtained (Fig. 2). Extracellular glucose could be transported into the cell by transporters such as Gtsc and Malk and then decomposes the resulting sugar under the action of the PTS system. Fructose-6-phosphate further enters the glycolytic pathway and is converted into pyruvate. At the same time, extracellular xylose enters the cell by the ribose transport system ATP-binding protein (Rbsa), ribose transport system substrate-binding protein (RbsB), and D-xylose transport system permease protein (XylH), and then under the action of xylose decomposition enzymes such as XylA and XylB, it is decomposed into xylulose 5-phosphate, which is converted into pyruvate through the pentose phosphate pathway. Pyruvate can also be catalyzed by pyruvate dehydrogenase to generate acetyl coenzyme A (acetyl-CoA), an intermediate product involved in the reaction of butyric acid and acetic acid. Acetyl-CoA generates acetylacetyl-CoA under the action of atoB and is then reduced to 3-hydroxybutyryl-CoA, which is further dehydrated into crotonyl-CoA, which is dehydrogenated to butyryl-CoA. The butyryl-CoA group is transferred from butyryl-CoA to acetic acid, and finally butyric acid is formed. In addition, acetyl-CoA is further metabolized to acetic acid by the action of enzymes pta and ackA, and acetic acid and acetyl-CoA can be converted to each other through the pathway, and acetic acid can further produce ethanol. In addition to acetic acid, pyruvate can also generate lactic acid through the catalyticaction of ldh. Moreover, Acetoacetate decarboxylase (EC: 4.1.1.4), which converts acetoacetate into acetone, were not annotated in the genome of BRM001. Thus, BRM001 might not be capable of produce actone and isopropanol.

#### Carbon and nitrogen substrate assimilation of BRM001

Based on the reconstruction of metabolic pathways, the assimilation metabolism of carbon substrates and nitrogen substrates and the synthesis of amino acids and vitamins were analyzed. The summary is shown in Additional file 1: Tables S4 and S5. The results showed that the carbohydrate assimilation pathways of glucose, xylose, fructose, mannose, galactose, cellobiose, and sucrose were complete in *C. beijerinckii* BRM001. It can be seen that BRM001 can use a variety of carbon sources, which is consistent with the previous report (Jiang et al. 2018).

The nitrogen assimilation pathways suggested that BRM001 can use nitrate, nitrite, ammonium salt, and ammonia. In addition, the sulfur utilization pathway in the metabolic pathway of the strain is complete. The biosynthesis pathways of tryptophan and biotin were incomplete, while the biosynthesis pathways of other amino acids and vitamins were complete. The amount of tryptophan will directly affect the rate of protein synthesis. Biotin is an essential growth factor for microorganisms. Biotin has a significant effect on cell metabolism, growth, and reproduction and is directly related to cell morphology and cell membrane permeability (Wu et al. 2016). Therefore, *C. beijerinckii* BRM001 cannot synthesize the two important biomass components, which may affect cell growth and limit butyric acid production.

To verify the results of KEGG pathway annotation, a single factor experiment was performed on the total synthetic medium for BRM001 growth. When tryptophan or biotin was excluded from the medium, cell growth could be detected. After removing tryptophan and biotin from the total synthetic medium, the growth of BRM001 was decreased by 7.8% and 73% compared with the control group, respectively, confirming that BRM001 has defects in the synthesis of the above two substances (Additional file 1: Fig. S1).

#### Application of *C. beijerinckii* genome annotation

Genome sequencing and annotation can reveal the metabolic characteristics of microorganisms, providing information for the design of microbial fermentation. In this study, the genetic profile and metabolic pathways for carbon, nitrogen, and essential nutrients of *C. beijerinckii* BRM001 were analyzed by the genome sequencing and KAAS annotation. The assimilation of carbon sources for BRM001 was explored, and a metabolic network for the production of butyric acid from glucose and xylose was constructed. The glucose and xylose were the main two carbohydrates in rape straw hydrolysate. The analysis of the utilization of carbon and nitrogen source materials could help us to purposefully screen the suitable substrates affecting the synthesis of butyric acid. The growth verification of the synthetic defect of tryptophan and biotin annotated in the genome annotation was performed. Tryptophan and biotin were further used as candidate additives for promoting the synthesis of butyric acid by BRM001. Thus, the genome annotation provides an important basis for fermentation regulation and the acquisition of candidate targets. The fermentation optimization strategy guided by the genome sequencing and annotation is more purposeful and effective.

### Selection of fermentation method

Two fermentation methods were used to produce butyric acid using rape straw, which were named step-by-step hydrolysis fermentation (liquid fermentation) and simultaneous enzymatic hydrolysis semi-solid fermentation (SEHSF). The yields of butyric acid in liquid fermentation and SEHSF were 6.75 ± 0.73 g/L and 6.57 ± 0.56 g/L, respectively. The yield difference was small, so SEHSF was used for the subsequent experiments. The production of butyric acid by SEHSF not only avoids the secondary pollution of the environment due to hydrolysis residue, but also reduces the process of filtration, making the whole process more economical. After semi-solid fermentation, the liquid product was butyric acid, and the fermentation residue was dried as a bacterial protein product. The protein contents before and after fermentation are shown in Table 2.

**Table 2:** Protein content in fermentation residue

	Crude protein content (%)	True protein content (%)	Acid-soluble protein content (%)
Unpretreated rape straw	3.47 ± 0.18	3.24 ± 0.13	1.37 ± 0.11
Pretreated rape straw	1.94 ± 0.17	1.34 ± 0.09	0.09 ± 0.01
fermentation residue	18.45 ± 0.28	15.84 ± 0.32	13.80 ± 0.19

### Optimization of butyric acid fermentation process based on genome annotation

#### Optimization of medium composition based on genome annotation

##### Determination of straw addition

The more the straw is added, the higher is the carbon substrate content in the fermentation medium. At the same time, with the increase of straw content, the content of inhibitors in the hydrolysate also increases, which might inhibit microbial fermentation. To investigate the effect of rape straw addition on the yield of butyric acid, 50 mL BRM001 seed liquid was inoculated into 100 mL of fermentation medium 2 with a straw content of 0, 10, 20, 30, 40, 50, 60,70, or 80 g/L. SEHSF was carried out at 35 °C for 9 days. The content of butyric acid in fermentation broth was determined. The results showed that the yield of butyric acid increased with the increase of straw addition. When the amount of straw added was 70 g/L, the yield of butyric acid was the highest, reaching 10.69 ± 0.17 g/L (Additional file 1: Fig. S2). Therefore, 70 g/L was selected for the follow-up experiment.

##### Selection of nitrogen source and determination of its addition

**Fig. 3:**
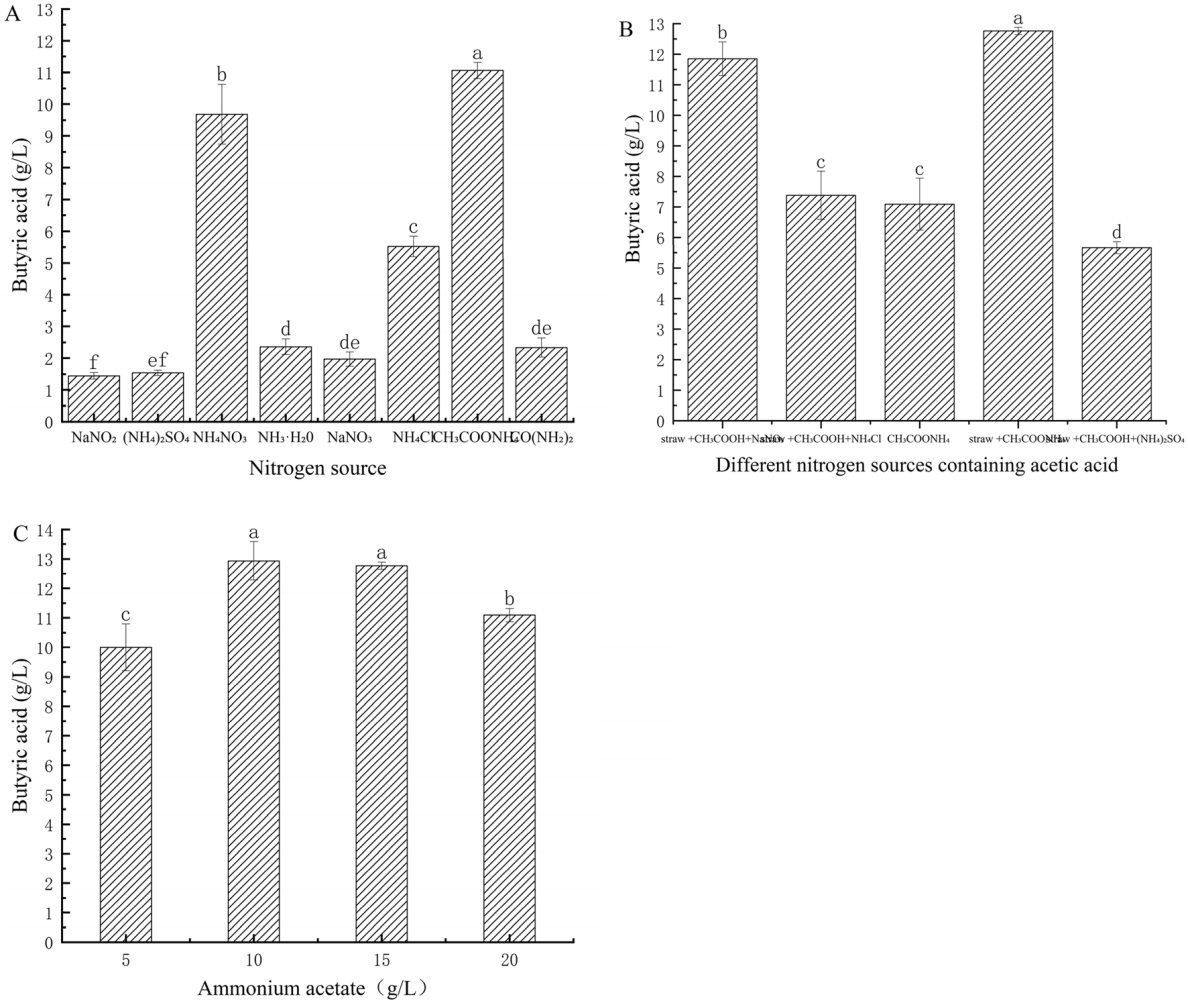
Determination of nitrogen source (**A** Effects of different nitrogen sources on butyric acid yield; **B** Effects of different nitrogen sources containing acetic acid on butyric acid production; **C** Effect of nitrogen source addition on butyric acid yield)

According to the KAAS annotation results, BRM001 could mainly use nitrate, nitrite, ammonium salt, and ammonia as the nitrogen sources. These nitrogen sources were added to the basic fermentation medium containing 70 g/L straw at a concentration of 10 g/L. The results are shown in Fig. 3A. It can be seen that ammonium nitrate and ammonium acetate had better effects, with butyric acid yields of 9.87 ± 0.65 g/L and 10.98 ± 0.89 g/L, respectively. Ammonium chloride ranks second, with a butyric acid yield of 5.46 ± 0.23 g/L. The effect of other nitrogen sources is poor, with butyric acid yields of less than 3 g/L. Therefore, ammonium acetate is the best inorganic nitrogen source. However, at high temperatures and under high pressure, ammonium nitrate may explode in the presence of oxidizable substances and electric sparks, which is not conducive to production safety (Han et al. 2023). In addition, studies have shown that *C. beijerinckii* NCIMB 8052 cultured in acetate highly expresses CoA transferase. In the presence of acetate, the specific activity of acetate kinase (ack) and butyrate kinase (buk) is also higher, cell growth is faster, and butyric acid production is increased (Chen and Blaschek 1999).

Therefore, ammonium acetate was selected as the nitrogen source for fermentation to produce butyric acid. However, ammonium acetate also introduced a carbon source while introducing a nitrogen source. To eliminate this effect, acetic acid was added with other ammonium salts and ammonium acetate was added as the only carbon and nitrogen source. It can be seen from Fig. 3B that acetic acid can indeed act as a carbon source to support butyric acid bacteria to produce acid, but in the absence of straw saccharification liquid, the content of carbon is not enough, and the yield of butyric acid is low. When the medium contains straw, acetic acid, and ammonium chloride or ammonium sulfate, the yield is still nearly 50% lower than that of the medium containing only straw and ammonium acetate. It can be seen that ammonium acetate is indeed the best nitrogen source and that the optimal amount is 10 g/L (Fig. 3C).

##### Single factor optimization of fermentation medium components

**Fig. 4:**
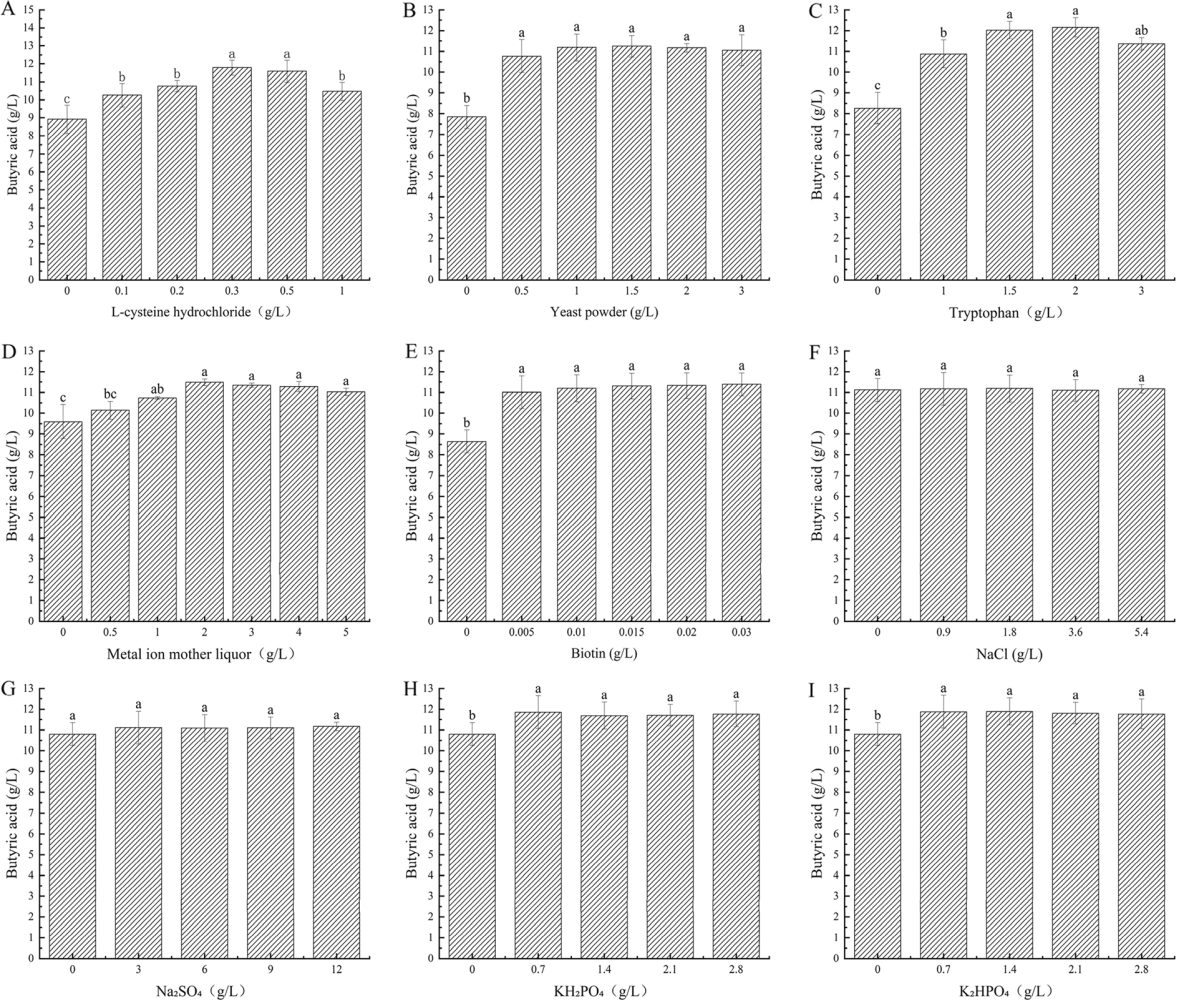
Effects of different fermentation conditions on butyric acid production by BRM001 (**A** L-cysteine hydrochloride, **B** yeast powder, **C** tryptophan; **D** metal ion mother liquor, **E** biotin, **F** NaCl, **G** NaSO_4_; **H** KH_2_PO_4_; **I** K_2_HPO_4_)

Based on basic fermentation medium 2 with 70 g/L straw powder and 10 g/L ammonium acetate, the amount of yeast powder, biotin, tryptophan, metal ion solution, and L-cysteine hydrochloride was changed in a single factor experiment. After 9 days of static fermentation at 35 °C, the content of butyric acid was measured. From Fig. 4, it can be concluded that the optimum amounts were 1 g/Lyeast powder, 0.01 g/Lbiotin, 2%metal ion solution, 2 g/Ltryptophan, and 0.3 g/L L-cysteine hydrochloride.

##### PB test and steepest climbing test

The PB method is widely used to optimize the composition of microbial fermentation medium. According to the above single factor experiment, straw, ammonium acetate, yeast powder, metal ion solution, tryptophan, biotin, and L-cysteine hydrochloride, which had significant effects on the yield of butyric acid, were selected. Each factor takes two levels, and the content of the intermediate in the product is the response value. The data were processed by Design-Export 8.0.6, and the importance of each factor was compared. The results showed that ammonium acetate, metal ion solution, and L-cysteine hydrochloride had significant effects, tryptophan had extremely significant effects, and the other factors were not significant. The regression equation of the model is as follows:

*Y* = 3.01 + 0.0251 *A + 0.0936*B + 0.0370*C + 0.0895*D + 0.1746*E + 0.0233*F − 0.1259*G. The *P* value of the overall model is 0.0152, which shows that the effect is significant and the model is statistically significant.

It can be seen from the equation that B, D, and E have positive effects and G has a negative effect. Therefore, the levels of B (ammonium acetate), D (metal ion solution), and E (tryptophan) increase and the level of F (L-cysteine hydrochloride) decreases.Finally, 11 g/Lammonium acetate, 1.7%metal ion solution, 1.7 g/Ltryptophan, and 0.3 g/L L-cysteine hydrochloride were obtained as the central values.

##### Response surface methodology

**Fig.5:**
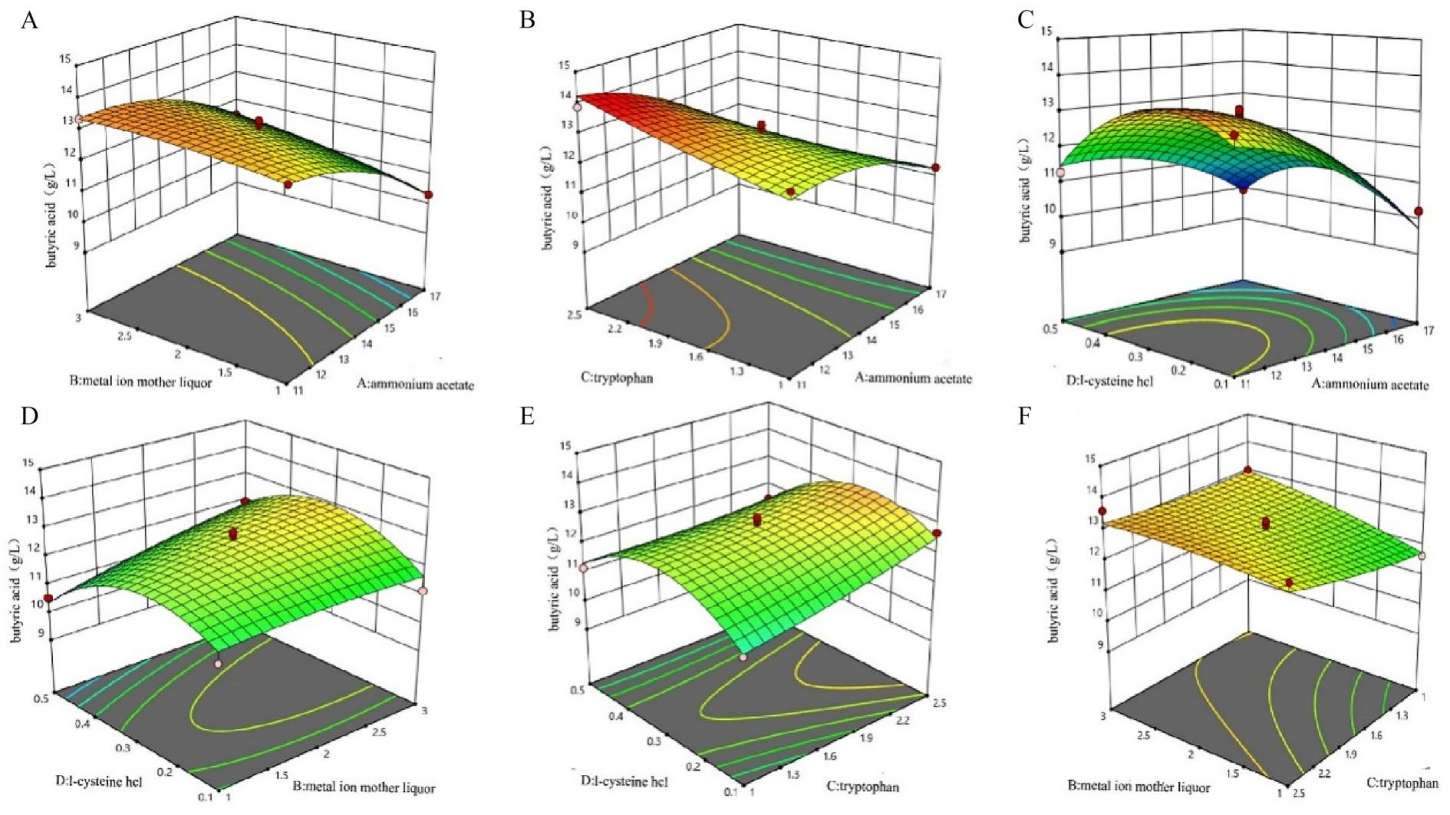
Response surface interaction diagram. (**A** metal ions and ammonium acetate; **B** tryptophan and ammonium acetate; **C** L-cysteine hydrochloride and ammonium acetate; **D** L-cysteine hydrochloride and metal ions; **E** L-cysteine hydrochloride and tryptophan; **F** Metal ions and tryptophan)

Using Design-Expert, the following regression equation was obtained: Y = 12.75 − 1.14*X_1_ + 0.3850*X_2_ + 0.3367*X_3_ + 0.2560*X_4_ + 0.2150*X_1_X_2_ + 0.4475*X_1_X_3_ + 0.3675*X_1_X_4_ + 0.2375*X_2_X_3_ + 0.3775*X_2_X_4_ − 0.3000*X_3_X_4_ − 0.5702*X_1_^2^ − 0.1377*X_2_^2^ + 0.0973*X_3_^2^ − 1.23X_4_^2^, where Y represents the content of butyric acid and X_1_, X_2_, X_3_, and X_4_ represent the amounts of ammonium acetate, metal ion solution, tryptophan, and L-cysteine hydrochloride, respectively. The F test was used to analyze whether the model is significant. The regression model was analyzed by variance analysis; we found *R*^2^ = 93.84%, and its fitting degree is good. The model *P* > 0.05, which indicates that the lack of fit is not significant relative to the absolute error, indicating that the regression equation fits well with the experimental results. The first terms X_1_ and X_2_ of the model are extremely significant, X_3_ and X_4_ are significant, the interaction term X_1_X_2_ is significant, and the rest are not significant. The quadratic terms X_1_^2^ and X_4_^2^ are extremely significant, and the rest are not significant. Figure 5A–F shows a three-dimensional response surface diagram of butyric acid with ammonium acetate, metal ion solution, tryptophan, and L-cysteine hydrochloride. It can be seen from Fig. 5A that when the content of tryptophan and L-cysteine hydrochloride is constant, the more the ammonium acetate is added, the greater the contribution of metal ion solution to acid production is.

Compared with the orthogonal experiment, RSM tests the influence of various factors and their interactions on the response value by establishing a surface model. It can continuously analyze each level of the test, significantly reduce the number of experiments, and shorten the test cycle. In the present study, the highest yield of butyric acid was predicted to be 13.8975 g/L with 11.16 g/L ammonium acetate, 2.99% metal ion solution, 2.44 g/L tryptophan, and 0.29 g/L L-cysteine hydrochloride. To facilitate the operation, the amount of ammonium acetate was modified to 11 g/L, the amount of metal ion solution was modified to 3%, the amount of tryptophan was modified to 2.5 g/L, and the amount of L-cysteine hydrochloride was modified to 0.3 g/L. The improved fermentation medium was used for verification, and three experiments were carried out. The content of butyric acid was 13.16 ± 0.46 g/L, which reached 95% of the predicted value of the model. The difference between the actual value and the predicted value is less than 1 g/L; this difference is relatively small, and the model has certain credibility.

#### Optimization of fermentation conditions

##### Single factor optimization of fermentation conditions

**Fig.6:**
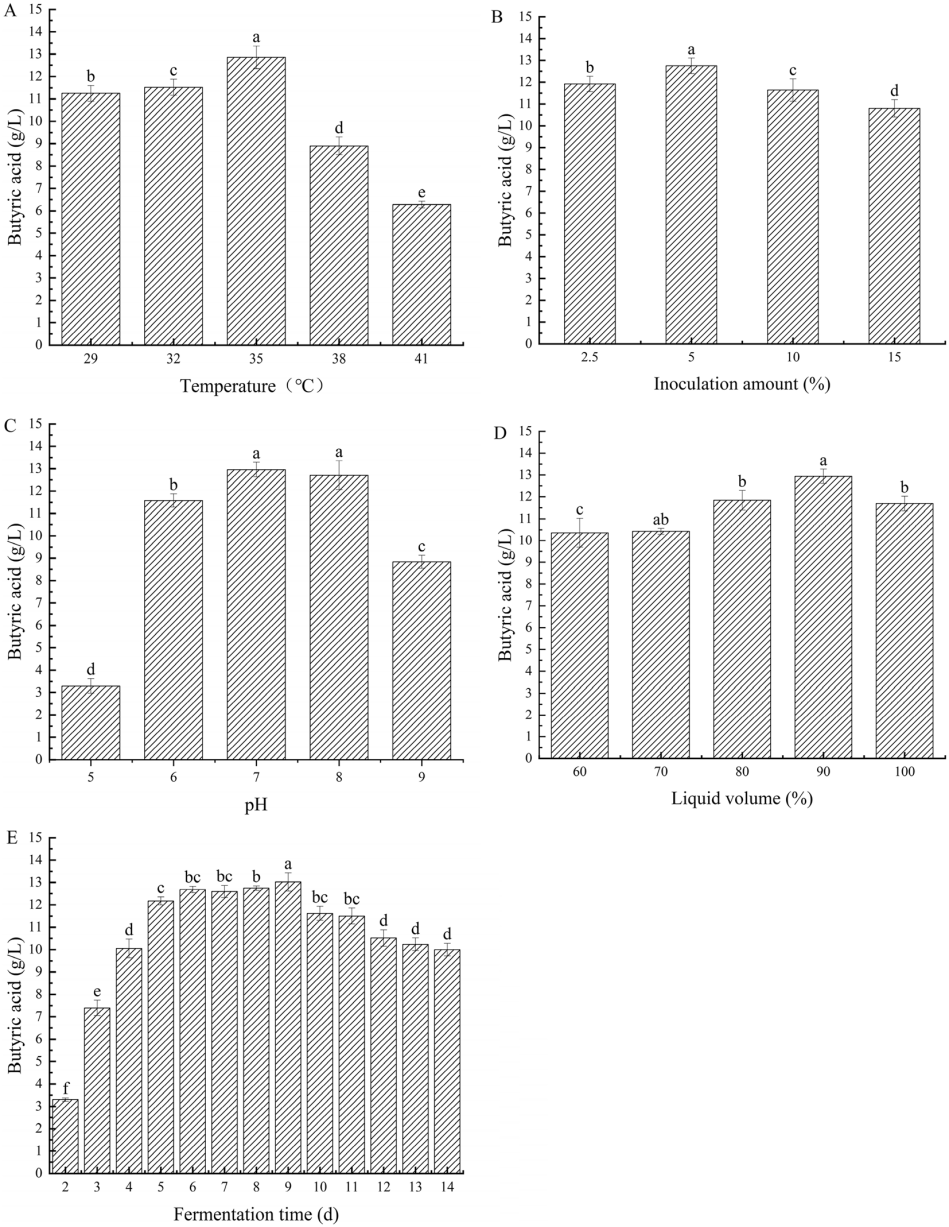
Effects of different fermentation conditions on butyric acid production by BRM001 (**A** temperature, **B** inoculum, **C** initial pH, **D** liquid volume, **E** fermentation time)

Based on the optimized fermentation medium of *C. beijerinckii* BRM001, the fermentation conditions of butyric acid production were optimized by a single factor experiment. The single factors were mainly inoculum size, temperature, initial pH, liquid volume, and fermentation time. Under the same culture conditions, the butyric acid content of *C. beijerinckii* BRM001 increased with the increase of temperature, inoculation amount, and liquid volume. When the temperature was 35 °C, the inoculation amount was 5%, and the liquid volume was 90%, the yield of butyric acid was the highest (Fig. 6). In addition, *C. beijerinckii* BRM001 exhibited better butyric acid production under neutral conditions. When the pH value was 7, the yield of butyric acid reached a peak of 12.96 ± 0.33 g/L; in the fermentation cycle of *C. beijerinckii* BRM001, the content of butyric acid showed an increasing trend within 0–9 days, reaching a peak of 13.02 ± 0.71 g/L at 9 days. The optimal fermentation time of *C. beijerinckii* BRM001 was determined to be 9 days, and the highest butyric acid yield was 13.02 ± 0.71 g/L. Finally, the inoculation amount was 5%, the temperature was 35 °C, the pH was 7, the liquid volume was 90%, and the fermentation time was 9 days as the orthogonal test value point.

##### Orthogonal test

According to the results of our single factor experiment, the factors that have great influence on yeast fermentation were selected to design an orthogonal experiment, and the fermentation conditions were further optimized. The initial pH, fermentation time, fermentation temperature, and inoculation amount were used as four experimental factors, and four levels were selected for the experiment. The orthogonal test design was carried out by an L_16_ (4^5^) orthogonal table. The results are shown in Table 3.

**Table 3:** Orthogonal test results and analysis

Factor level	A Temperature (℃)	B Inoculation amount (%)	C Time (d)	D pH	Vacant column	Butyric acid (g/L)
1	26	2.5	6	6.5	1	10.33 ± 0.23
2	26	5	7	7	2	10.94 ± 0.33
3	26	7.5	8	7.5	3	11.24 ± 0.27
4	26	10	9	8	4	11.04 ± 0.43
5	29	2.5	7	7.5	4	11.81 ± 0.26
6	29	5	6	8	3	12.47 ± 0.32
7	29	7.5	9	6.5	2	12.98 ± 0.36
8	29	10	8	7	1	13.24 ± 0.64
9	32	2.5	8	8	2	11.88 ± 0.63
10	32	5	9	7.5	1	12.83 ± 0.63
11	32	7.5	6	7	4	12.97 ± 0.25
12	32	10	7	6.5	3	11.38 ± 0.21
13	35	2.5	9	7	3	13.80 ± 0.73
14	35	5	8	6.5	4	13.77 ± 0.64
15	35	7.5	7	8	1	12.86 ± 0.53
16	35	10	6	7.5	2	13.55 ± 0.29
K1	10.887	11.955	12.330	12.115	12.315	
K2	12.625	12.503	11.748	12.738	12.338	
K3	12.625	12.512	12.532	12.358	12.223	
K4	13.495	12.303	12.663	12.063	12.398	
R	2.608	0.557	0.915	0.675	0.175	

As shown in Table 4, the effects of A (temperature), B (inoculation amount), C (time), and D (pH) on butyric acid content were ordered as follows: A > C > D > B. According to the k value, the optimal combination of culture conditions was A_4_B_3_C_4_D_2_. According to the variance analysis (Table 4), the effect of inoculation amount and pH on the concentration of butyric acid was not significant (*P* > 0.05), the effect of temperature was extremely significant (*P* < 0.01), and the effect of fermentation time was significant (*P* < 0.05). In conclusion, the following optimized fermentation conditions were obtained by our orthogonal experiment: initial pH, 7; inoculation amount, 7.5%; temperature, 35 °C; and fermentation time, 9 days.

**Table 4:** The analysis of variance

Model	Sum of squares	Degree of freedom	Mean square	F	Significance
Modified model	17.557^a^	12	1.463	19.266	0.016*
Intercept	2391.944	1	2391.944	31497.981	0.000**
A	14.450	3	4.817	69.430	0.003**
B	0.608	3	0.203	2.669	0.221
C	2.292	3	0.764	10.060	0.045*
D	0.206	3	0.069	0.905	0.532
Error	0.228	3	0.076		
Total	2409.728	16			
Corrected total	17.784	15			

*significant (p < 0.05 ); **very significant (p < 0.01)

^a^R square = 0.987 (adjusted a. R square = 0.936)

##### Verification test

Verify the best fermentation conditions obtained by orthogonal experiment. The yield of butyric acid increased from 13.16 ± 0.46 g/L to 13.89 ± 0.72 g/L, which was 1.06 times higher than that before optimization.

#### Non-sterile fermentation of butyric acid

Although there have been many reports on the microbial production of butyric acid on a pilot scale, in all of them, fermentation was performed under sterile conditions (Ai et al. 2016; Jiang et al. 2018). Many studies also introduced nitrogen to maintain an anaerobic environment during fermentation, which increases the costs and hinders industrialization.

To apply the above optimization results to actual production, taking into account the convenience of industrial application, we increased the fermentation volume to 500 mL, 1000 mL, and 2500 mL for raw material fermentation under non-sterile conditions to produce butyric acid. The results are shown in Table 5. It can be seen that both under sterile conditions and in raw material fermentation, the fermentation volume of 100 mL was slightly better than that of 2.5 L; in both small-scale and large-scale fermentation, the yield of butyric acid produced under sterile conditions and by raw material fermentation is not much different; at the four scales carried out in the present study, the difference was less than 1%, and the yield of butyric acid reached about 13.5 g/L. This represents one of the highest butyric acid yields ever reported in batch fermentation without the use of large-scale precision fermentation equipment, without the use of engineered strains, and our yield is only slightly lower than the yield obtained by Fonseca team's (14.9 g/L butyric acid from sugarcane straw) (Fonseca et al. 2021). However, the cost of microwave pretreatment and sterile bioreactor fermentation is much higher than that of this study (Fonseca et al. 2021). At the same time, the fermented waste residue can be used as a bacterial protein feed after drying, which makes full use of the value of the fermented waste residue and does not produce secondary pollution, making the whole process environmentally friendly and economical.

**Table 5:** Effects of scale-up and unsterilized fermentation on butyric acid production

Fermentation method	Fermentation scale (mL)	Butyric acid (g/L)
Sterilization fermentation	100	13.86 ± 0.77
	500	13.76 ± 0.53
	1000	13.66 ± 0.37
	2500	13.52 ± 0.87
Un-sterilized fermentation	100	13.80 ± 0.53
	500	13.74 ± 0.70
	1000	13.69 ± 0.46
	2500	13.42 ± 0.83

In this experiment, even if the non-sterile fermentation medium was inoculated with *C. beijerinckii* for SEHSF, the yield of butyric acid was almost the same as that of butyric acid fermented under sterile conditions, and the fermentation scale was appropriately expanded. The yield of butyric acid was almost unchanged. This process is suitable for industrial butyric acid production by rape straw fermentation and provides a theoretical basis for the industrialization of butyric acid production by biomass fermentation.

## Conclusion

In this study, a non-sterile semi-solid fermentation approach for butyric acid production from rape straw was carried out and the yield of butyric acid was 13.42 ± 0.83 g/L in a 2.5-L fermentor, which showed a certain industrial potential. This study may provide theoretical and technical support for the large-scale use of microbial fermentation of lignocellulose to produce butyric acid and promote the industrialization of microbial fermentation of bio-based raw materials to produce butyric acid.

### Supplementary Information

**Additional file 1:** Main reagents.

## Data Availability

The datasets supporting the conclusions of this article are included within the article and its additional file. Data will be made available on request.

## Abbreviations

KAAS: Kyoto Encyclopedia of Genes and Genomes Automatic Annotation Server
KEGG: Kyoto Encyclopedia of Genes and Genomes
NCBI: National Center for Biotechnology Information
RCM: Reinforced Clostridial Medium
RSM: Response surface methodology
SEHSF: Simultaneous enzymatic hydrolysis semi-solid fermentation
SSF: Simultaneous saccharification and fermentation
