# Supplementary material for: Optimization of Clostridium beijerinckii semi-solid fermentation of rape straw to produce butyric acid by genome analysis
Source: Bioresour Bioprocess. 2024 Feb 14;11(1):24. doi: 10.1186/s40643-024-00742-y (PMC10992193; doi:10.1186/s40643-024-00742-y)
Supplement: Supplementary file 1 — Additional file 1: Main reagents. [file 40643_2024_742_MOESM1_ESM.docx]

Table S1 Main reagents

| Reagent Name | Grade | Manufacturer |
| --- | --- | --- |
| Sodium acetate anhydrous | Analytically pure (AR) | China Pharmaceutical Group Chemical Co., Ltd, China |
| Magnesium sulfate heptahydrate | Analytically pure (AR) | China Pharmaceutical Group Chemical Co., Ltd, China |
| Soluble starch | Biochemical pure (BR) | China Pharmaceutical Group Chemical Co., Ltd, China |
| Dipotassium phos-phate | Analytically pure (AR) | Beijing Oberstar Biotechnology Co., Ltd, China |
| Potassium dihydrogen phosphate | Analytically pure (AR) | Beijing Oberstar Biotechnology Co., Ltd, China |
| Citric acid monohydrate | Analytically pure (AR) | Beijing Oberstar Biotechnology Co., Ltd, China |
| Sodium citrate dihydrate | Analytically pure (AR) | Beijing Oberstar Biotechnology Co., Ltd, China |
| 3,5-dinitrosalicylic acid | Analytically pure (AR) | Beijing Oberstar Biotechnology Co., Ltd, China |
| Yeast powder | Analytically pure (AR) | Beijing Oberstar Biotechnology Co., Ltd, China |
| Sodium hydroxide | Analytically pure (AR) | Beijing Oberstar Biotechnology Co., Ltd, China |
| Anhydrous glucose | Analytically pure (AR) | Chengdu Cologne Chemical Co., Ltd, China |
| Sodium chloride | Analytically pure (AR) | Chengdu Cologne Chemical Co., Ltd, China |
| Liquid paraffin | Analytically pure (AR) | Chengdu Cologne Chemical Co., Ltd, China |
| Biotin | Analytically pure (AR) | Shanghai Aladdin Biochemical Technology Co., Ltd, China |
| l-cysteine hcl | Analytically pure (AR) | Shanghai Aladdin Biochemical Technology Co., Ltd, China |
| Novozymes cellulaseCellic CTec3 HS | - | Novens Biotechnology Co., Ltd, China |
| 0.22μm organic phase filter membrane | - | UNICO (Shanghai) Scientific Instruments Co., Ltd, China |

Table S2 Main instruments and equipment

| Equipment name | Model | Manufacturer |
| --- | --- | --- |
| Acidometer | PHS-3C | Chengdu Century Ark Technology Co., Ltd, China |
| Medical centrifuge | TG-16 | Sichuan Shuke Instrument Co., Ltd, China |
| Culturecase of anaerobe | HYQX-Ⅲ | Shanghai Yuejin Medical Device Co., Ltd, China |
| Thermostat water bath | HH-4 | Shanghai Lichen Bangxi Instrument Technology Co., Ltd, China |
| Biochemical incubator | LRH-300 | Changzhou Nokia Instrument Co., Ltd, China |
| Visible spectrophotometer | V-1000 | Aoyi Instrument Co., Ltd, China |
| Benchtop | W-CJ-2D | Shanghai Sujing Industrial Co., Ltd, China |
| Automatic kjeldahl nitrogen analyzer | K9840 | Jinan Haineng Instrument Co., Ltd, China |
| Graphite digestion instrument | SH220F | Jinan Haineng Instrument Co., Ltd, China |
| Gas chromatograph | 5977B-7890B | Agilent Technology Co., Ltd，American |
| Vertical mode steam sterilizer | LS-50HJ | Jiangyin Binjiang Medical Equipment Co., Ltd, China |

Table S3: Inhibitor concentration of straw saccharification liquid

| Name | content (%) |
| --- | --- |
| Acetic acid | 0.992 |
| Ferulic acid | 0.005 |
| 4-hydroxybenzaldehyde | 0.011 |
| Syringaldehyde | 0.002 |
| 5-hydroxymethyl furaldehyde | 0.012 |
| Phenol | 0.079 |
| Furfuraldehyde | 0.006 |
| Acetosyringone | 0.003 |

Table S4 Nutrient Utilization of BRM001

|  | Nutritional ingredient | Whether it can be utilized |
| --- | --- | --- |
| Carbon source | Glucose | + |
|  | Xylose | + |
|  | Fructose | + |
|  | Mannose | + |
|  | Starch | + |
|  | Galactose | + |
|  | Sugar | + |
|  | Cellobiose | + |
| Nitrogen source | Nitrogen | + |
|  | Hydrazine | - |
|  | Nitronitrogen | + |
|  | Hydroxylamine | + |
|  | Nitrite | + |
|  | Nitrate | + |
|  | Urea | - |
|  | Ammonia water | + |
|  | Nitrogen oxide | - |
| Sulphur source | Sulfate | + |
|  | Sulfite | + |
|  | Sulfides | + |
|  | Thiosulfate | - |
|  | Taurine | - |

Note: ' + ' means can be used, ' - ' means can’t be used

Table S5 Analysis of KAAS synthesis ability of nutrients

|  | Nutritional ingredient | Whether it can be synthesized |
| --- | --- | --- |
| Amino acid | Valine | + |
|  | Leucine | + |
|  | Isoleucine | + |
|  | Lysine | + |
|  | Arginine | + |
|  | Phenylalanine | + |
|  | Tyrosine | + |
|  | Tryptophan | - |
|  | Glycine | + |
|  | Alanine | + |
|  | Methionine | + |
|  | Proline | + |
|  | Serine | + |
|  | Tyrosine | + |
|  | Cysteine | + |
|  | Aspartic acid | + |
|  | Threonine | + |
|  | Glutamic acid | + |
|  | Histidine | + |
|  | Asparagine | + |
|  | Glutamine | + |
| Vitamin | Thiamin | + |
|  | Riboflavin | + |
|  | Nicotinic acid | + |
|  | Pantothenic acid | + |
|  | Folic acid | + |
|  | Biotin | - |

Note: ' + ' means it can be synthesized, ' - ' means it cannot be synthesized.

Table S6 Genes in the butyric acid biosynthesis pathway of *C. beijerinckii*

| Symbol | Name | Enzyme |
| --- | --- | --- |
| pgi | glucose-6-phosphate isomerase | EC:[5.3.1.9](https://www.genome.jp/entry/5.3.1.9) |
| pfk | ATP-dependent phosphofructokinase / diphosphate-dependent phosphofructokinase | EC:2.7.1.11;2.7.1.90 |
| fbaA | fructose-bisphosphate aldolase, class II | EC:4.1.2.13 |
| gapA | glyceraldehyde 3-phosphate dehydrogenase (phosphorylating) | EC:1.2.1.12 |
| pgk | phosphoglycerate kinase | EC:2.7.2.3 |
| gpmA | 2,3-bisphosphoglycerate-dependent phosphoglycerate mutase | EC:5.4.2.11 |
| gpmI | 2,3-bisphosphoglycerate-independent phosphoglycerate mutase | EC:5.4.2.12 |
| pyk | pyruvate kinase | EC:2.7.1.40 |
| ldh | L-lactate dehydrogenase | EC:1.1.1.27 |
| nifJ | pyruvate-ferredoxin/flavodoxin oxidoreductase | EC:1.2.7.1 |
| atoB | acetyl-CoA C-acetyltransferase | EC:2.3.1.9 |
| pta | phosphate acetyltransferase | EC:2.3.1.8 |
| ackA | acetate kinase | EC:2.7.2.1 |
| acyP | acylphosphatase | EC:3.6.1.7 |
| paaH | 3-hydroxybutyryl-CoA dehydrogenase | EC:1.1.1.157 |
| crt | enoyl-CoA hydratase | EC:4.2.1.17 |
| atoA | acetate CoA/acetoacetate CoA-transferase beta subunit | EC:2.8.3.9;2.8.3.8 |
| fabV | enoyl-[acyl-carrier protein] reductase / trans-2-enoyl-CoA reductase (NAD+) | EC:1.3.1.9;1.3.1.44 |
| tktA | transketolase | EC:2.2.1.1 |
| talA | transaldolase | EC:2.2.1.2 |
| xylA | xylose isomerase | EC:5.3.1.5 |
| xylB | xylulokinase | EC:2.7.1.17 |
| wecB | UDP-N-acetylglucosamine 2-epimerase (non-hydrolysing) | EC:5.1.3.14 |
| prsA | ribose-phosphate pyrophosphokinase | EC: 2.7.6.1 |
| gtsA | glucose/mannose transport system substrate-binding protein | K17315 |
| gtsC | glucose/mannose transport system permease protein | K17317 |
| malK | multiple sugar transport system ATP-binding protein | K10111 |
| rbsA | ribose transport system ATP-binding protein | K10441 |
| rbsB | ribose transport system substrate-binding protein | K10439 |
| xylF | D-xylose transport system substrate-binding protein | K10543 |
| xylH | D-xylose transport system permease protein | K10544 |
| aas | Acyl CoAligase-coupled transporters | 4.C.1.1.16 |


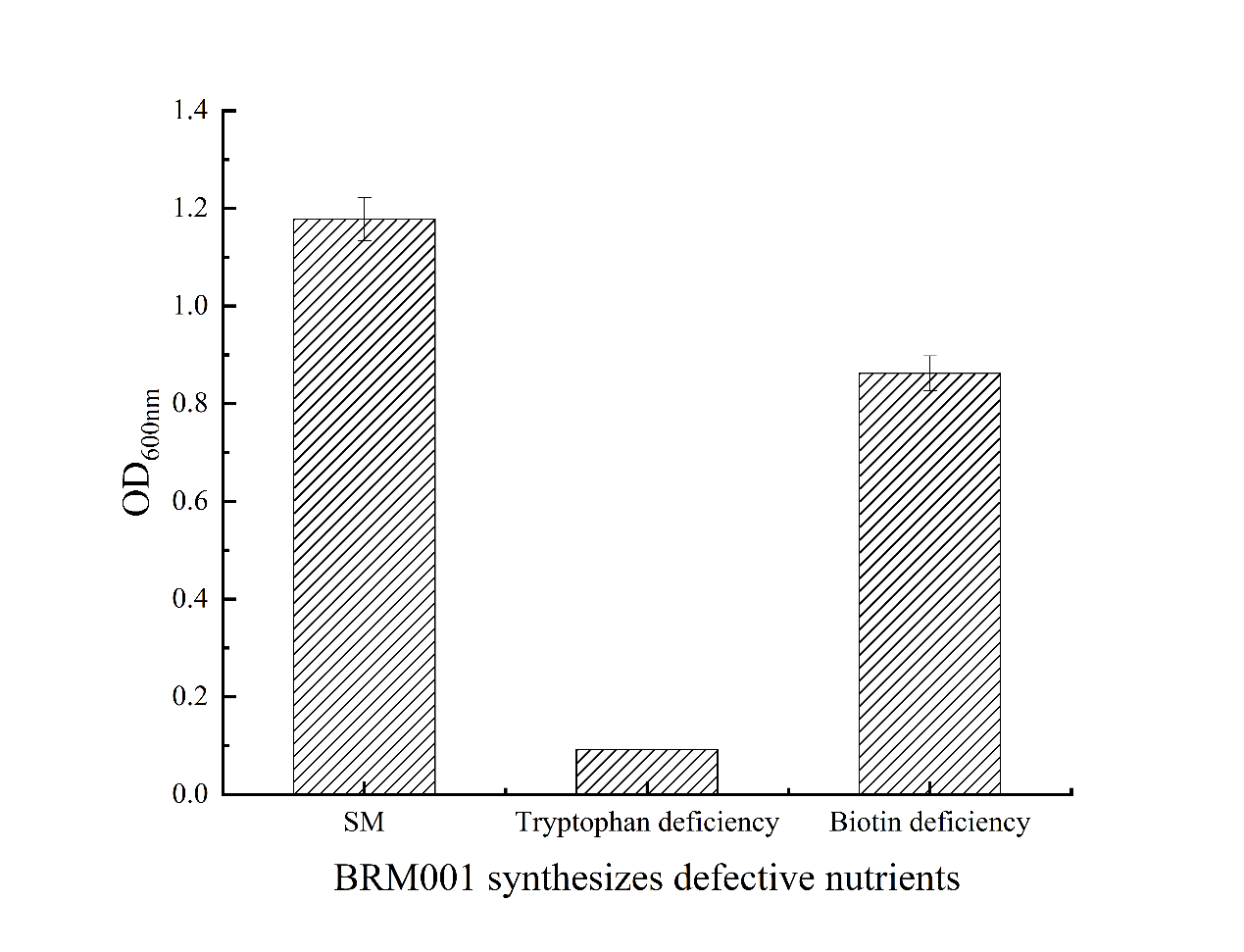


Fig. S1 Single-factor growth experiments for *C. beijerinckii* BRM001.


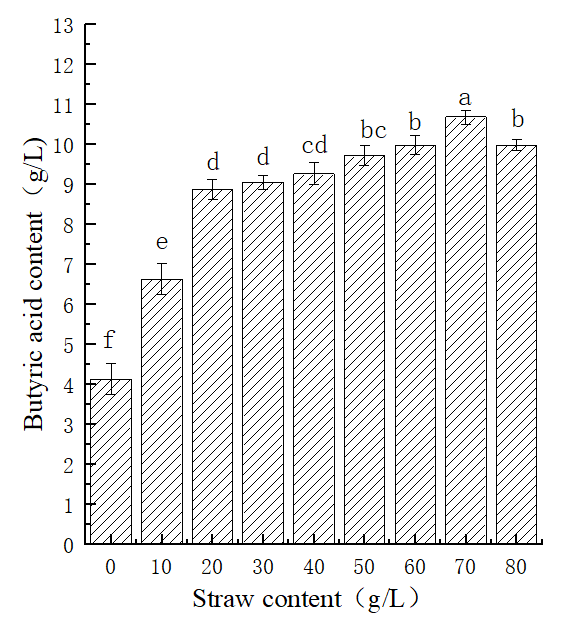


Fig. S2 Effect of straw addition on butyric acid yield
